# Reply to Hall et al. Comment on “Dinesen et al. Diphoterine for Chemical Burns of the Skin: A Systematic Review. *Eur. Burn J.* 2023, *4*, 55–68”

**DOI:** 10.3390/ebj4030026

**Published:** 2023-06-29

**Authors:** Felicia Dinesen, Pernille Pape, Martin Risom Vestergaard, Lars Simon Rasmussen

**Affiliations:** 1 Department of Anaesthesia, Centre of Head and Orthopaedics, Section 6011 Rigshospitalet, Copenhagen University Hospital, DK-2100 Copenhagen, Denmark; 2 Department of Clinical Medicine, University of Copenhagen, DK-2200 Copenhagen, Denmark

We thank Hall et al. [1] for their interest in our article *Diphoterine for chemical burns of the skin: A systematic review* [2]. We appreciate their enthusiasm for evidence in the field of treating chemical lesions. We agree that our findings should be interpreted with caution because of the limited number of studies and the relatively low number of patients included in those investigations.

New treatments should be carefully tested before implementation in the treatment of patients and blind, randomized trials can provide important evidence for a beneficial effect; however, conducting such trials can be a huge challenge, especially in emergency situations and it can be tempting to look at other types of studies. We decided to include studies with a comparator, both randomized trials and observational studies, but we think it is less relevant to include case reports and case series without a comparator. 

It would also be problematic to consider studies that are entirely focusing on biomarkers or mediators because of the indirectness associated with surrogate measures [3].

Clinicians have to consider numerous factors when deciding how chemical burns should be treated, such as time to treatment, the amount of rinsing solution needed, how the product can be stored and transported, and the risk of hypothermia. The use of Diphoterine certainly offers some theoretical advantages in addition to the reduction in pain and effect on pH detected in our review. This should be assessed in prospective studies that should probably be multicenter trials because chemical lesions are not that common.
